# Structural, spectroscopic and cytotoxicity studies of TbF_3_@CeF_3_ and TbF_3_@CeF_3_@SiO_2_ nanocrystals

**DOI:** 10.1007/s11051-013-1958-x

**Published:** 2013-09-05

**Authors:** Tomasz Grzyb, Marcin Runowski, Krystyna Dąbrowska, Michael Giersig, Stefan Lis

**Affiliations:** 1 Department of Rare Earths, Faculty of Chemistry, Adam Mickiewicz University, Grunwaldzka 6, 60-780 Poznan, Poland; 2 Bacteriophage Laboratory, Institute of Immunology and Experimental Therapy, Polish Academy of Sciences, Rudolfa Weigla 12, 53-114 Wrocław, Poland; 3 Institute of Experimental Physics, Freie Universität Berlin, Arnimallee 14, 14195 Berlin, Germany

**Keywords:** Nanoparticles, Core/shell, Silica, Luminescence, Rare earth fluorides, Cytotoxicity

## Abstract

**Abstract:**

Terbium fluoride nanocrystals, covered by a shell, composed of cerium fluoride were synthesized by a co-precipitation method. Their complex structure was formed spontaneously during the synthesis. The surface of these core/shell nanocrystals was additionally modified by silica. The properties of TbF_3_@CeF_3_ and TbF_3_@CeF_3_@SiO_2_ nanocrystals, formed in this way, were investigated. Spectroscopic studies showed that the differences between these two groups of products resulted from the presence of the SiO_2_ shell. X-ray diffraction patterns confirmed the trigonal crystal structure of TbF_3_@CeF_3_ nanocrystals. High resolution transmission electron microscopy in connection with energy-dispersive X-ray spectroscopy showed a complex structure of the formed nanocrystals. Crystallized as small discs, ‘the products’, with an average diameter around 10 nm, showed an increase in the concentration of Tb^3+^ ions from surface to the core of nanocrystals. In addition to photo-physical analyses, cytotoxicity studies were performed on HSkMEC (Human Skin Microvascular Endothelial Cells) and B16F0 mouse melanoma cancer cells. The cytotoxicity of the nanomaterials was neutral for the investigated cells with no toxic or antiproliferative effect in the cell cultures, either for normal or for cancer cells. This fact makes the obtained nanocrystals good candidates for biological applications and further modifications of the SiO_2_ shell.

**Graphical Abstract:**

.
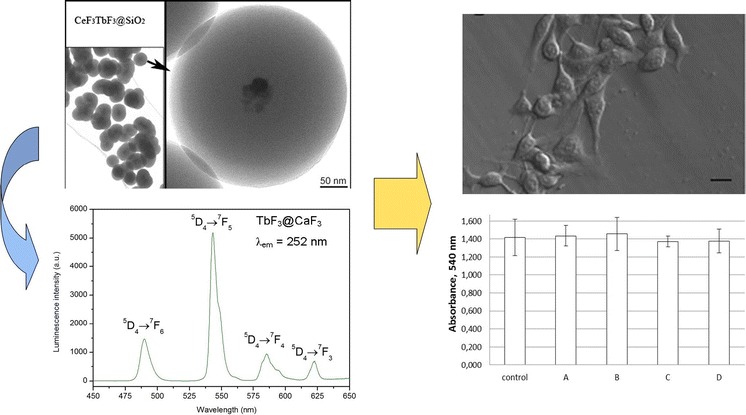

## Introduction

Luminescent nanocrystals (NCs) activated by lanthanide ions (Ln^3+^) have been the object of intense research in recent years as a direct consequence of their unique properties (Grzyb and Lis 2011; Grzyb et al. 2012a, b; Wang et al. 2006a, 2010, 2011a; Wiglusz et al. 2009; Yan and Yan 2008; Yang et al. 2011). Taking advantage of phenomenon such as energy transfer (ET) and the characteristic features of *f*-electronic transitions, it is possible to obtain efficient luminescence from Ln^3+^-doped materials even when the materials are prepared at nanodimensions. This property is necessary for their potential applications in many areas. The current development of high technologies is one example of domains requiring appropriately designed systems (Bedekar et al. 2009; Boulon 2012). Furthermore, advanced fields of science, such as medicine and biology, use characteristic properties of Ln^3+^-doped NCs for bioimaging or cancer therapy (Bao et al. 2011; Di et al. 2011a; Diamente et al. 2006; Kang et al. 2011; Ren et al. 2012). Lanthanide-doped nanomaterials are attractive in connection with their exceptional optical and chemical properties, i.e. large effective Stokes shifts, high resistance to photobleaching, blinking and photochemical degradation. These excellent luminescent properties of the lanthanide ions, long luminescence lifetimes and the ability of ET processes occurring between dopants, make it possible to obtain an intense, visible emission under UV or IR (up-conversion) irradiation. In addition, the emission (luminescence lifetime) from Ln^3+^ is usually longer than the fluorescence of tissue (background) and differs in the spectral region, which allows for time-resolved fluorescence lifetime imaging (TRFI) (Cubeddu et al. 2002; Väisänen et al. 2000). This avoids disadvantages of UV excitation resulting in auto-fluorescence of cells. Hence, they present interesting alternatives for dye-based markers. Functionalization and coating of Ln^3+^-doped materials could increase their impact on fundamental biomedical research and clinical practice. Also receiving nanoparticles with mesoporous structure allows for designing multifunctional systems for simultaneous drug delivery and cell imaging (Di et al. 2011b; Janowski et al. 2012; Park et al. 2012).

Rare earth (RE) fluorides are important hosts for luminescent Ln^3+^ ions (Chen et al. 2010a; Diamente et al. 2006; Evanics et al. 2006; Kang et al. 2011; Wang et al. 2006a; Yan and Yan 2008). The low phonon energy of these compounds (e.g. LaF_3_, phonon energy is close to 350 cm^−1^) is the reason for their high effectiveness as matrices for displaying the luminescence of Ln^3+^ ions. The known methods of RE fluoride synthesis consist of a precipitation reaction in various systems, a thermal decomposition reaction of trifluoroacetate precursors in high boiling solvents, or a combination of these methods (Diamente et al. 2006; Evanics et al. 2006; Kang et al. 2011; Wang et al. 2006a; Yan and Yan 2008). Cerium fluoride is an especially important host for Tb^3+^ ions promoting efficient ET between Ce^3+^ and Tb^3+^ (Chai et al. 2009; Grzyb et al. 2012a; Wang et al. 2006b). ET results in an increased luminescence intensity which is important for bio-photonic applications. In this way lowering of the concentration of NCs used in in vitro imaging is possible; however, the luminescent signal can still be satisfactory. The presence of trivalent lanthanide ions in NCs could also change their magnetic properties. Ln^3+^ ions are known for their paramagnetic properties which can be used for magnetic resonance imaging (Grzyb et al. 2012c; Johnson et al. 2011). Also Ce^3+^ ions are known for their paramagnetic properties (Chen et al. 2010b).

Recently, the core/shell structures have been intensely investigated, and much valuable information such as the changed luminescence intensity or biological compatibility was obtained (Abel et al. 2009; Fu and Sun 2009; Ghosh et al. 2008; Kang et al. 2011; Zhu et al. 2008). The increased interest concerning core/shell structures is an effect of their improved physicochemical properties attributed to the surface coating, higher thermal stability and enhanced water solubility (Ghosh et al. 2008; Wang et al. 2009).

Silica coating is a popular method for improving water solubility and protecting NC cores from the surrounding environment (Runowski et al. 2012). Inexpensive and easy methods of obtaining spherical particles with narrow size distributions, chemical inertness and optical transparency are some of the advantages of using SiO_2_ as shells in NCs. Increased optical response, higher luminescence and longer decay times have been observed when the cores of luminescent Ln^3+^-doped materials are covered by SiO_2_ shells (Wang et al. 2009). As a result, biological effects of silica nanoparticles have become the subject of growing interest in recent years (Wang et al. 2012).

Developing core/shell nanostructures is also important for optoelectronic applications. Moreover, silica-coated NCs have become increasingly important in biomedical research (Bao et al. 2011; Hu et al. 2011). Because of the low chemical activity of SiO_2_, the coating process seems to be an effective way of lowering the cytotoxicity of the cores. However, it must nonetheless be mentioned that SiO_2_-based NCs could still be toxic, especially if their size is small (Lin et al. 2006; Yang et al. 2010). The HaCaT cells viability could be significantly decreased after exposure for SiO_2_ nanoparticles, which also induced apoptosis in a size-dependent manner (Yang et al. 2010). The mechanism underlying the toxic effects of SiO_2_ nanoparticles is based on the abnormal expression of oxidative stress-associated molecules, which inhibit cell growth and cause apoptosis (Lin et al. 2006; Yang et al. 2010). Effects of particle size on the cytotoxicity were also noticed in the case of silver nanoparticles. Smaller nanoparticles (20 nm) were more toxic than larger ones (80 and 110 nm) (Park et al. 2011). The most pronounced effects of these nanoparticles were a changed cellular metabolic activity and membrane damage (Park et al. 2011).

Covering NCs with a SiO_2_ shell allows for their further modification. The presence of the –OH groups in the SiO_2_ shell is an advantage over unmodified cores. These groups could be used for chemical modifications and conjugation of biomolecules such as biotin, antibodies and oligonucleotides (Selvan et al. 2009). The silica shell also improves the water solubility of the NCs, and is known as an anti-agglomerating agent. Therefore, it is important to study the effect of SiO_2_ coatings on the spectroscopic properties of NC cores. The aim of this research was to investigate this effect. Our experiments have been additionally extended to be able to study the cytotoxicity of the prepared materials.

## Experimental

Reagents: Tb_4_O_7_ (99.99 %, Stanford Materials) was dissolved in ultra-pure nitric acid, HNO_3_ (POCh, Gliwice, Poland) in order to obtain Tb(NO_3_)_3_. Cerium chloride, CeCl_3_·6H_2_O (Sigma-Aldrich, 99.9 %), ammonium fluoride NH_4_F (POCh S.A., ACS grade, 98 %), Triton X-100 (POCh, Gliwice, Poland, reagent grade), TEOS (Sigma-Aldrich, 98 % reagent grade), 25 % NH_4_OH aqueous solution (Chempur, pure p.a.) and ethanol C_2_H_5_OH (99.98 % reagent grade) were used without further purification. For all experiments distilled water was used.

### Synthesis of TbF_3_@CeF_3_ nanoparticles

In our experiment, the core/shell, TbF_3_@CeF_3_ nanocrystals were formed spontaneously from Tb^3+^- to Ce^3+^-containing solutions. This self-organization resulted in the synthesis of trigonal nanoplates with a Tb^3+^-rich core and a Ce^3+^-rich shell.

In order to synthesize TbF_3_@CeF_3_, solution A was prepared as follows: an aqueous solution of Tb(NO_3_)_3_ and CeCl_3_ was mixed in the molar ratio 15:85, respectively. The obtained solution was mixed with 1.25 g of Triton X-100 (surfactant) and filled with water up to 50 mL. Subsequently, solution B containing the fluoride ion source was prepared as follows: 0.83 g of NH_4_F (50 % excess) was dissolved in water and transferred onto a hot-plate magnetic stirrer. Then, 5 g of Triton X-100 was added to this solution and filled with water up to 100 mL. The whole system was stirred and heated up to 50 °C. This temperature was maintained over the entire process of synthesis. Afterwards, solution A was added drop by drop to the continuously stirred and heated solution B. Addition of solution A lasted approximately 30 min. After this time the reaction was finished, and a white precipitate of TbF_3_@CeF_3_ NCs was obtained. The precipitate was purified by centrifugation and washed several times with water and ethanol. The product exhibited a bright green luminescence under UV lamp irradiation (*λ*_ex_ = 254 nm).

### Synthesis of TbF_3_@CeF_3_@SiO_2_

**Fig. 1:**
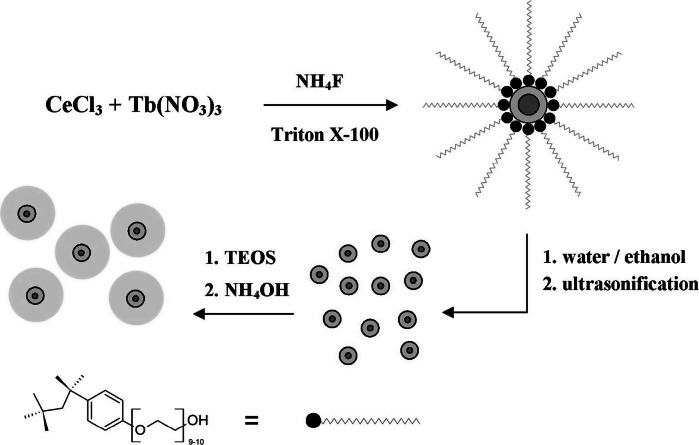
Schematic presentation of the formation process of TbF_3_@CeF_3_ and TbF_3_@CeF_3_@SiO_2_ nanoparticles

Twenty milligram of the previously synthesized TbF_3_@CeF_3_ nanocrystals was dispersed in 10 mL of water. Subsequently, the colloid was mixed with 180 mL of ethanol and 10 mL of 25 % aqueous solution of ammonia, NH_4_OH. The final colloid was ultrasonicated in order to fully homogenise the whole system. The homogeneous colloidal solution was transferred onto the magnetic stirrer and vigorously stirred, at ambient conditions. Afterwards, 1.5 mL of TEOS was injected into this system. The reaction (TEOS hydrolysis) was carried out over 2 h. When the reaction was finished, the clear and transparent solution became white and turbid. The trigger of this was the formation of the silica shell. The hybrid, core/shell type product was purified by centrifugation and washed several times with water and ethanol. The final complex, core/shell type product, exhibited also a bright green luminescence under UV lamp irradiation (*λ*_ex_ = 254 nm); however, its intensity was less than in the case of TbF_3_@CeF_3_ NCs. The scheme of synthesis is presented in Fig. 1.

## Characterisation

X-ray diffraction patterns (XRD) were measured with a Bruker AXS D8 advance diffractometer using Cu Kα radiation (*λ* = 1.541874 Å) in the 2*θ* ranges from 6° to 60°. The IR absorption spectrum was recorded between 500 and 4,000 cm^−1^ on an FTIR spectrophotometer, Bruker FT-IR IFS 66/s. The material was mixed with KBr and then pressed into discs. The hydrodynamic diameter and *ζ*-potential of the obtained nanoparticles in water were measured with a Malvern Nano Zeta Sizer, Dynamic Light Scattering (DLS) instrument (He–Ne laser 633 nm, Max 5 mW). The excitation and emission spectra as well as luminescence lifetime measurements were performed on a Hitachi F-7000 fluorescence spectrophotometer at room temperature. Excitation and emission spectra were corrected for the instrumental response. The measured luminescence decays showed nonexponential character, which is a result of cross-relaxation between Tb^3+^ ions. The kinetics of the cross-relaxation is complex, and the model explaining this process has been previously reported (Ricci et al. 2010). However, the decay profiles can be fitted with an exponential function as a first approximation to the reported kinetics model. Therefore, to calculate luminescence lifetimes of TbF_3_@CeF_3_ NCs, luminescence decays were fitted to an exponential function:

1 $$ I = I_{0} + A \cdot e^{{ - \frac{t}{\tau }}} $$

where *I* represents the intensity at any time, *I*_0_ is the intensity at *t* = 0 and *τ* is the luminescence lifetime. Nanocrystals covered by SiO_2_ shells, TbF_3_@CeF_3_@SiO_2_ showed different characteristics in their luminescence decays which were fitted by biexponential functions:

2 $$ I = I_{0} + A_{1} e^{{ - \frac{t}{{\tau_{1} }}}} + A_{2} e^{{ - \frac{t}{{\tau_{2} }}}} $$

Luminescence lifetimes were calculated with help of OriginLab 8.5 software. The goodness of fit to the time traces was not lower than *R* ^2^ = 0.996, and errors were not higher than *τ*_err_ < 0.01 ms. Transmission electron microscopy (TEM) was performed with a Philips CM200FEG electron microscope operating at 200 kV, equipped with an EDAX analyzer for qualitative and quantitative materials identification. The samples were dispersed on a Cu grid covered with 550-nm thick amorphous carbon film. Low-dose electron-beam imaging was used to prevent any beam damage of the sample and any additional heating effect of the electron beam (Henglein and Giersig 1999). The quality of the lattice images was improved using the conditions of minimum phase contrast according to Kunath (1987).

### The cytotoxicity of TbF_3_@CeF_3_ and TbF_3_@CeF_3_@SiO_2_ nanoparticles

The cytotoxicity of the investigated nanocrystals was tested in proliferation assays and by cell imaging. Both cancer cells and normal cells were used. The HSkMEC (Human Skin Microvascular Endothelial Cells) cell line was obtained from Cell Culture Collection of the IIET (Wroclaw, Poland) (Crola Da Silva et al. 2009). The B16F0 mouse melanoma cancer cell line was obtained from the American Type Culture Collection (ATCC, USA). Both lines are maintained in the Cell Culture Collection of the IIET (Wroclaw, Poland). Cells were cultured in OptiMEM (Invitrogen, Cergy Pontoise, France) supplemented with 5 % foetal bovine serum, 40 μg/mL gentamycin (Invitrogen) and 0.05 μg/mL fungizone (Invitrogen). Cells were seeded at 2 × 10^4^ cells/cm^2^ at 96-well plate or 6-well plate 24 h before experiments and maintained at 37 °C in a 5 % CO_2_/95 % air atmosphere during the pre-culture and the experiment. Final concentrations of the nanoparticles were: 0.05 mg/mL (0.029 mg/cm^2^) and 0.005 mg/mL (0.0029 mg/cm^2^) for TbF_3_@CeF_3_@SiO_2_ and its mass-equivalents for TbF_3_@CeF_3_ (the mass-equivalents represent the dose of uncoated-TbF_3_@CeF_3_ contained in TbF_3_@CeF_3_@SiO_2_). Higher concentrations of NCs caused mechanical damage to cell cultures and difficulties in interpreting the cytotoxicity analysis. The formed colloids were not stable, and the NCs tended to agglomerate. Before cell incubation, the prepared colloids were sterilized. Control cultures were supplemented with PBS.

The effect of nanoparticles on the cells was assessed as follows: (i) cell condition and morphology was assessed by optical microscopy in sequential imaging, (ii) total cell production was assessed by SRB assay (sulphorodamine B assay). The details of this technique were described by Skehan et al. (1990). The assay was performed after 72-h exposures of the cultured cells to the tested agents. The cells attached to the plastic were fixed by gently layering cold 50 % TCA (trichloroacetic acid, Aldrich-Chemie, Germany) on the top of the culture medium in each well. The plates were incubated at 4 °C for 1 h and then washed five times with tap water. The background optical density was measured in the wells filled with the culture medium, without the cells. The cellular material fixed with TCA was stained with 0.4 % sulforhodamine B (SRB, Sigma, Germany) dissolved in 1 % acetic acid (POCh, Gliwice, Poland) for 30 min. The unbound dye was removed by rinsing with 1 % acetic acid. The protein-bound dye was extracted with 100 μL 10 mM unbuffered Tris base (POCh, Gliwice, Poland) for the determination of the optical density (at 540 nm) in a computer-interfaced, 96-well microtiter plate reader Multiskan RC photometer (Labsystems, Helsinki, Finland). Each nanoparticle type and concentration in each cell line culture was tested two times in six-well groups (*N* = 6).

## Results and discussion

### Structure and morphology

Synthesis of the nanocrystalline rare earth fluorides is usually based on a precipitation reaction. It can be carried out under various conditions, such as reverse micelles formed in surfactants containing organic solvents, where the source of F^−^ ions can be a salt such as NaF or NH_4_F (Karbowiak et al. 2005; Qiu 2000) There are also other well-known methods, e.g. a decomposition of trifluoroacetates in high boiling solvents or solvo- and hydrothermal methods. However, these methods usually require an advanced laboratory, specific reagents and a long purification process (Guo 2006; Zhang et al. 2005). A simpler way of synthesizing monodispersed NCs is the co-precipitation method. In the presence of the chosen organic compounds, the growth and nucleation process of the precipitated crystals occurred, which results in specific nanodimensions. The role of a capping agent is often attributed to citric acid, ethylenediaminetetraacetic acid, polyethyleneimine or polyethylene glycol (Qiu et al. 2011; Wang et al. 2011b). For the synthesis of TbF_3_@CeF_3_ NCs we have used the co-precipitation method in the presence of the nonionic surfactant Triton X-100. Prepared in this way NCs were covered by a SiO_2_ shell, and core/shell structures were formed.

**Fig. 2:**
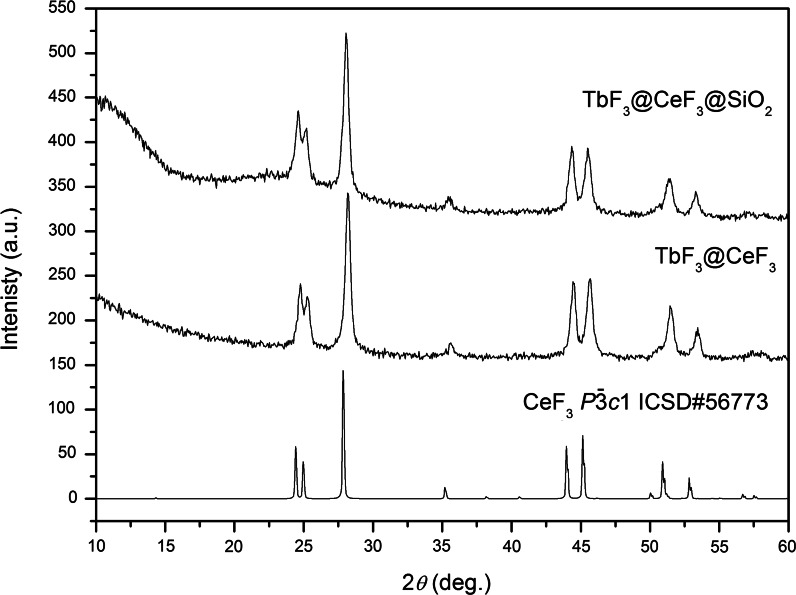
XRD patterns of TbF_3_@CeF_3_ and core/shell TbF_3_@CeF_3_@SiO_2_ nanocrystals

Cerium fluoride crystallizes in a trigonal crystal system with the space group $P\bar{3}c1$ 1(*D*_3*d*_) (Carnall et al. 1989; Cheetham et al. 1976). The Ce^3+^-ion in CeF_3_ crystals is coordinated by nine F^−^ anions and has a *C*_2_ site symmetry (Carnall et al. 1989). XRD patterns presented in Fig. 2, measured for the synthesized materials, fit well with standards from the ICSD database (Inorganic Crystal Structure Database) no 56773. Small nanoparticle sizes are responsible for the broadening of measured peaks. The broad band partially visible around 10° 2*θ* comes from amorphous silica. The characteristics of the pattern after surface covering is almost unchanged, which confirms that the applied process is safe for TbF_3_@CeF_3_ cores and does not influence their crystal structure.

The presence of a different phase is not visible in the XRD, which suggests that the TbF_3_ cores have the same structure as the CeF_3_ shell. The complex composition of the prepared product could also be responsible for the broadening of peaks in the XRD. Terbium fluoride usually crystallizes in the orthorhombic crystal structure with the space group *Pnma*. However, the displayed XRD patterns excluded the presence of phases other than trigonal CeF_3_. The explanation of this observation involves the manner in which these nanocrystals were synthesized. The precipitation of a product from the solution containing both lanthanide ions: Ce^3+^ and Tb^3+^, causes the formation of mixed fluorides containing both ions in the structure. The larger Ce^3+^ ions, present in the precipitated core, forced the formation of the trigonal structure.

**Fig. 3:**
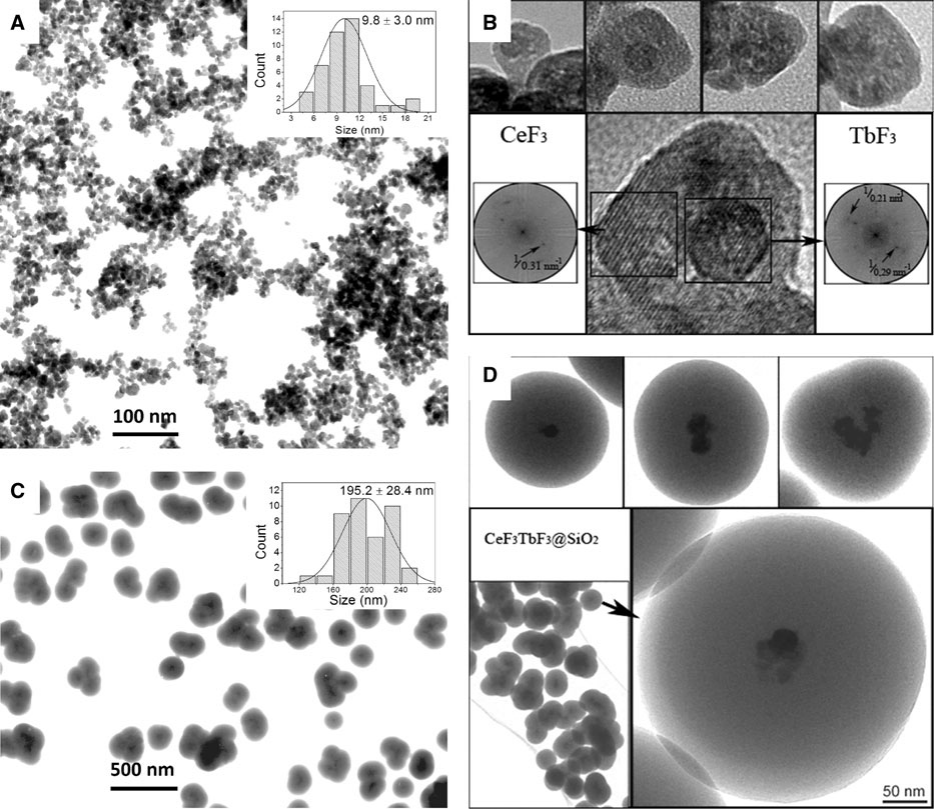
TEM and HR-TEM images of TbF_3_@CeF_3_ (**a**,**b**) and TbF_3_@CeF_3_@SiO_2_ core/shell nanoparticles (**c**,**d**)

Transmission electron microscopy was used for the imaging of the synthesized NCs and analysis of their morphology. Figure 3a shows TbF_3_@CeF_3_ fluoride NCs. Their sizes do not exceed 25 nm. Slight amounts of Triton X-100 molecules adsorbed on their surface suppress the formation of large agglomerates; however, they could still be observed. The formation of core/shell structures with a central core inside of the NCs, shown in Fig. 3d, confirms that occasionally the formed agglomerates are relatively weakly linked and could be broken due to the short ultrasonication. What is interesting, and remains unexpected, is the fact that in HR-TEM, the formation of the core/shell structure of these fluoride NCs results in a TbF_3_-rich core and a CeF_3_-dominated shell (Fig. 3b). Fourier transformation clearly indicates that both structures vary by differences in their lattice parameters. This situation is common in the obtained nanomaterial, which has been checked by HR-TEM. The reason for the crystallization mechanism leading to the presented structures is unknown. Most probable is that during the first stage of crystallization; when NH_4_F is added at concentrations lower than stoichiometric, Tb^3+^-rich nanoparticles are precipitated due to their lower solubility in the synthesis medium. Then, as additional reagent was being added, the Tb^3+^-rich nanoparticles acted as crystallization cores for the Ce^3+^-rich second phase. In fact, both phases crystallized in the $P\bar{3}c1$ crystal system, and the presence of more than a single phase in the XRD patterns was not observed. Also the luminescence analysis and the occurrence of ET confirmed that Ce^3+^ and Tb^3+^ must be present in the same phase.

The subject of spontaneous organization of nanosystems can be found in the literature. Some examples are: the spontaneous self-organization of Cu_2_O/CuO core–shell nanowires from copper nanoparticles or the organization of single CdTe nanoparticles into luminescent nanowires (Ji et al. 2010; Tang et al. 2002). Also, individual nanocrystals can assemble into more complex structures (Grzyb et al. 2013).

Figure 3c, d shows silica-covered-TbF_3_@CeF_3_@SiO_2_ nanoparticles. Their structure is well presented in HR-TEM and consists of small fluoride cores and relatively large silica shells with thicknesses of around 100 nm. The cores of the nanoparticles shown are composed of one to several TbF_3_@CeF_3_ nanocrystals.

**Fig. 4:**
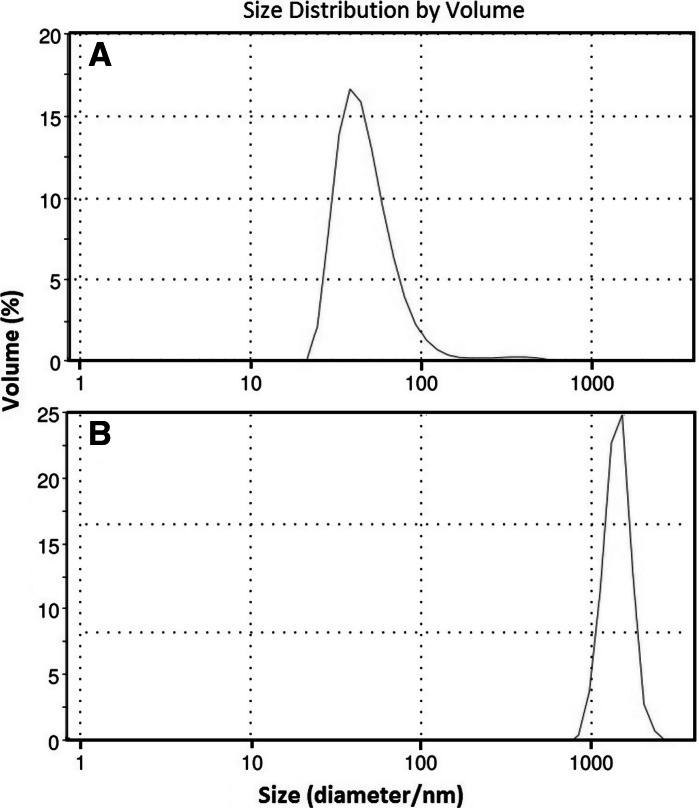
Particles size distribution obtained from DLS measurements; TbF_3_@CeF_3_ (**a**), TbF_3_@CeF_3_@SiO_2_ (**b**)

The average size distribution of the synthesized core and core/shell nanoparticles and their zeta potential (*ζ*-potential) were measured using the DLS technique. The measured *ζ*-potential of the TbF_3_@CeF_3_ was +22.7 mV, and −29.8 mV for TbF_3_@CeF_3_@SiO_2_ nanoparticles. The measured values are in good agreement with our predictions and literature data for similar compounds. According to the literature, the isoelectric point (point of zero charge) for LnF_3_ nanoparticles is around pH = 6, and for silica, (SiO_2_)_*n*_ particles, is ~pH = 3 (Philipse et al. 1994; Bogdan et al. 2011). The measured *ζ*-potential values for our samples (at pH = 5) indicate that after silica coating, there is a dramatic change in the *ζ*-potential, from a positive value +22.7 mV (protonated surface, e.g. LnOH_2_^+^) to a negative value −29.8 mV (deprotonated silica surface, –SiO^−^). The average size distribution of the obtained nanostructures (Fig. 4) was approximately 49 nm for TbF_3_@CeF_3_ (a), and approx. 1,411 nm for TbF_3_@CeF_3_@SiO_2_ (b). These values are larger than the average size calculated from TEM images, because the hydrodynamic diameter of the analysed particles is larger than their real size (size of the individual nanoparticles), which is common in such DLS measurements (Bogdan et al. 2011). Additionally, the TbF_3_@CeF_3_@SiO_2_ nanostructures tend to form agglomerates, whose hydrodynamic diameter registered during the experiment is obviously much larger compared to individual core/shell type nanoparticles.

**Fig. 5:**
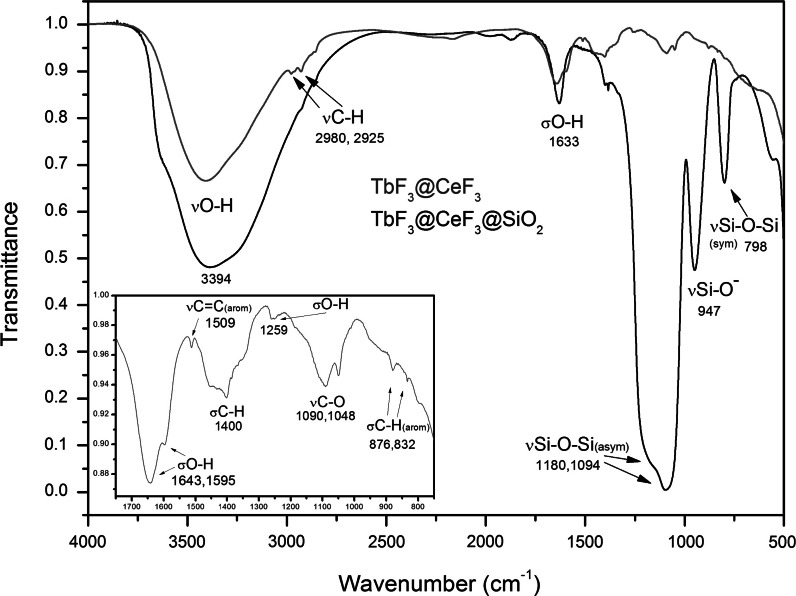
FT-IR absorption spectra of TbF_3_@CeF_3_ and TbF_3_@CeF_3_@SiO_2_ NCs

The synthesized NCs were examined by FT-IR spectroscopy in order to reveal the presence of Triton X-100 molecules on the NCs surface and silica in the core/shell type structure (Fig. 5). In the presented region of the infrared spectra there are no absorption peaks originating from Ln-F vibrations because they exhibit absorption at smaller wavenumbers, which is typical for inorganic fluorides. Both spectra show the most intense and broad absorption peak at around 3,394 cm^−1^, corresponding to O–H stretching vibrations of H_2_O (hydrogen-bonded water molecules) and O–H stretching vibrations of Triton X-100 molecules. In the case of the core/shell spectrum, this band is even more intense in comparison to the core, because of the overlapping of the O–H stretching vibrations from the previous molecules and silica (silanol groups, Si–OH). At 1,643, 1,633 and 1,595 cm^−1^ absorption peaks from the same molecules were registered and labelled as deformation vibrations of the O–H groups. The weak absorption bands at 2,980 cm^−1^ and 2,925 cm^−1^ were assigned to C–H stretching vibrations corresponding to the –CH_3_ and –CH_2_ groups of Triton X-100. Other peaks were labelled and assigned to the stretching (*ν*) and deformation (*σ*) vibrations corresponding to molecules of the surfactant, namely: *ν*C=C(arom) (1,509 cm^−1^), *σ*C–H (1,400 cm^−1^), *σ*O–H (1,259 cm^−1^), *ν*C–O (1,090, 1,048 cm^−1^) and *σ*C–H (arom) (876, 832 cm^−1^). Only in the spectrum of the core/shell NCs, three intense and dominant peaks originating from silica were observed and assigned to: *ν*Si–O-Si(asym) (1,180, 1,094 cm^−1^), *ν*Si–O-(947 cm^−1^) and *ν*Si–O-Si(sym) (798 cm^−1^). The presence of silica in the case of a core/shell type compound was confirmed. We also assumed that Triton X-100 molecules were adsorbed on the surface of the obtained NCs.

### Luminescence properties

The synthesized NCs showed intense green luminescence under UV irradiation (254 nm) corresponding to the electronic transitions of the Tb^3+^ ions. This luminescence can be used for typical optoelectronic applications such as various types of displays or lightening. However, the range of potential applications could be extended by the imaging of cells in fluorescence or confocal microscopy. The relatively small size of the particles allows them to penetrate cell membranes or to join to their surfaces. Silica covering displays a promising method for increasing the biocompatibility and gives the possibility of modifying the surface of inorganic NCs. However, according to the luminescence studies described below, the emission intensity of the core is highly influenced by the presence of a silica shell.

**Fig. 6:**
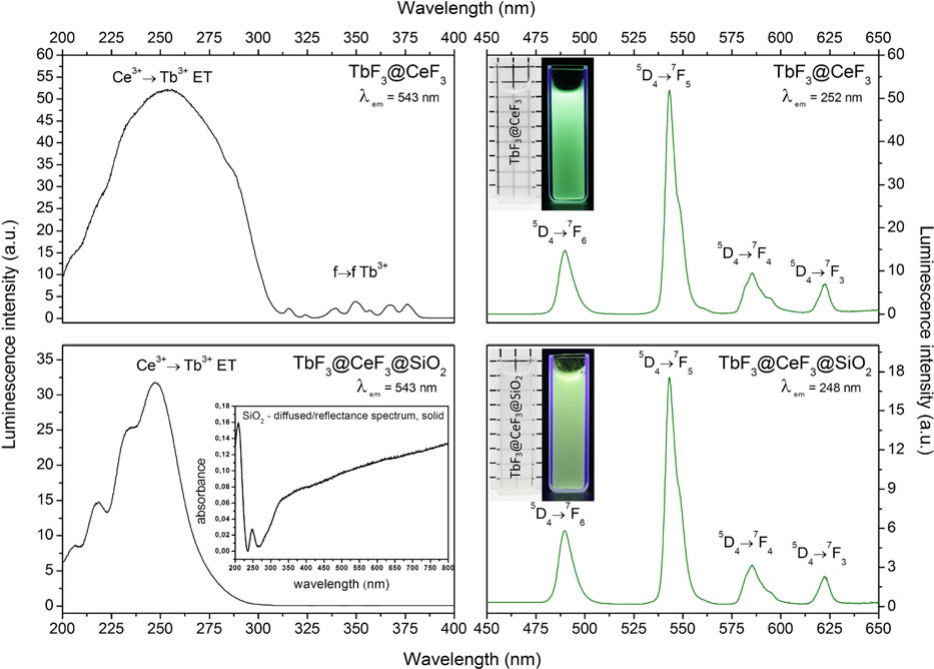
Excitation and emission spectra of TbF_3_@CeF_3_ and TbF_3_@CeF_3_@SiO_2_ nanoparticles, and absorption spectrum of pure SiO_2_ as an* inset* (*λ*_ex_ TbF_3_@CeF_3_ = 252 nm, *λ*_ex_ TbF_3_@CeF_3_@SiO_2_ = 248 nm, *λ*_em_ = 543 nm)

Figure 6 shows the excitation and emission spectra of the obtained nanoparticles. The excitation spectra of both materials show broad and intense bands with a maximum around 250–260 nm. These broad and intense bands could be observed as a result of the 4*f* ^1^ → 4*f* ^0^5*d* ^1^ transition of the Ce^3+^-ion (Guo et al. 2011). The presence of this broad band is evidence of ET between the Ce^3+^ and Tb^3+^ ions. Narrow and less intense bands in the range of 320–400 nm are connected with direct excitation of Tb^3+^ ions and are hindered by selection rules of *f*–*f* transitions. The covering of the TbF_3_@CeF_3_ NCs by silica changed the excitation spectra due to the absorption of UV light by SiO_2_ in this region (inset in Fig. 6). This effect is important, and therefore TbF_3_@CeF_3_@SiO_2_ showed much less effective luminescence than TbF_3_@CeF_3_. The emission spectra of the obtained NCs are typical for Tb^3+^-doped materials and are composed of narrow bands related to the *f*–*f* transitions of this ion.

Both obtained materials can easily form stable colloidal solutions, which are presented in Fig. 6. Despite the similar emission spectral characteristics, the colloidal solutions of the core and the core/shell NCs showed slightly different colours of emission, which is due to SiO_2_ light scattering and absorption at the UV wavelengths. The emission intensity is an important factor; however, the toxicity for cells can be much more critical for bioimaging applications and therefore should be taken as the priority in the design of nanolabels.

**Fig. 7:**
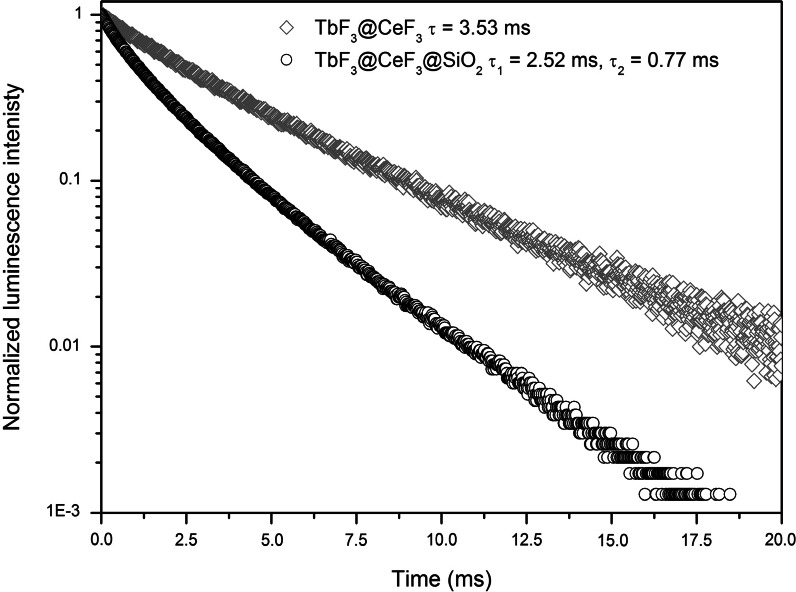
Luminescence lifetimes of TbF_3_@CeF_3_ and TbF_3_@CeF_3_@SiO_2_ nanoparticles (*λ*_ex_ TbF_3_@CeF_3_ = 252 nm, *λ*_ex_ TbF_3_@CeF_3_@SiO_2_ = 248 nm, *λ*_em_ = 543 nm)

The lower luminescence intensity of TbF_3_@CeF_3_@SiO_2_ is caused by the absorption of silica in the UV region, which results in a decreased excitation intensity. However, the measured luminescence decays, shown in Fig. 7, also indicate the formation of quenching centres after covering with silica. The FT-IR spectra suggest the presence of water molecules and, which seems even more possible, –OH groups in the SiO_2_ shell, which quenched the exited states of Tb^3+^ and additionally lowered the luminescence intensity of the core/shell type NCs. The calculated luminescence lifetimes are typical for Tb^3+^ ions, and the presence of the biexponential character of decays, in the case of the SiO_2_ covered NCs, could be explained by the two different surrounding environments of Tb^3+^ ions. Luminescence of the ions placed on the surface of NCs is quenched by –OH groups present in the SiO_2_ structure. Also the presence of a larger number of TbF_3_@CeF_3_ NCs incorporated into SiO_2_ could cause changes in the luminescence decay kinetics.

The disadvantages of SiO_2_ coating are undeniable and visible in the spectroscopic analysis presented. However, the same shell allows for the modification of NCs surface in a greater manner than uncoated particles. The receiving and designing of such nanostructures requires a compromise between a reduction in their luminescent properties and an expansion of their application possibilities.

**Fig. 8:**
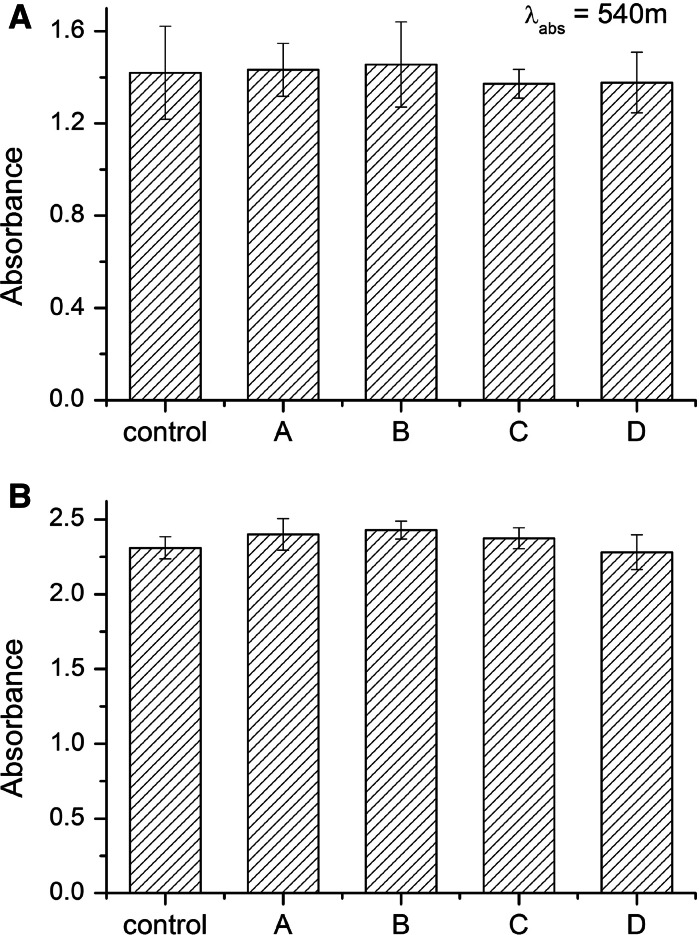
Cell viability in TbF_3_@CeF_3_ or TbF_3_@CeF_3_@SiO_2_ nanoparticle treated cell cultures (SRB assay). **a** B16F0 cells, **b** HSkMEC cells. Control-cells treated with PBS, A-cells treated with 0.05 mg/mL TbF_3_@CeF_3_@SiO_2_, B-cells treated with 0.005 mg/mL TbF_3_@CeF_3_@SiO_2_, C-cells treated with 0.01 mg/mL TbF_3_@CeF_3_ (mass equivalent for 0.05 mg TbF_3_@CeF_3_@SiO_2_) D-cells treated with 0.001 mg/mL TbF_3_@CeF_3_ (mass equivalent for 0.05 mg TbF_3_@CeF_3_@SiO_2_)

**Fig. 9:**
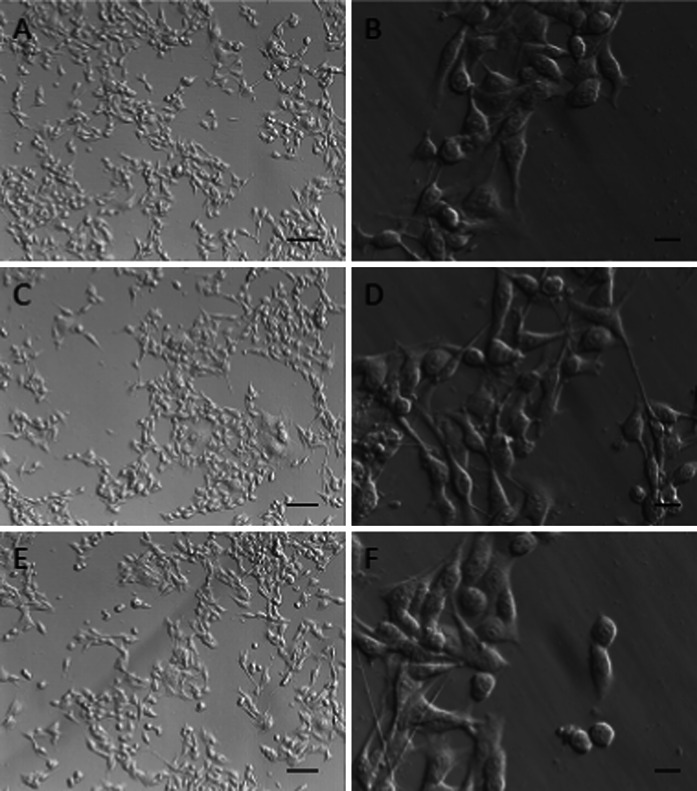
Microscopy images of B16F0 cells treated with TbF_3_@CeF_3_ or TbF_3_@CeF_3_@SiO_2_ nanoparticles (*scale bars* at **a**, **c** and **e**: 40 μm, *scale bars* at **b**, **d** and **f**: 10 μm). **a** and **b**: control cells. **c** and **d**: cells treated with TbF_3_@CeF_3_ for 72 h, washed before imaging. **e** and **f**: cells treated with TbF_3_@CeF_3_@SiO_2_ for 72 h, washed before imaging

**Fig. 10:**
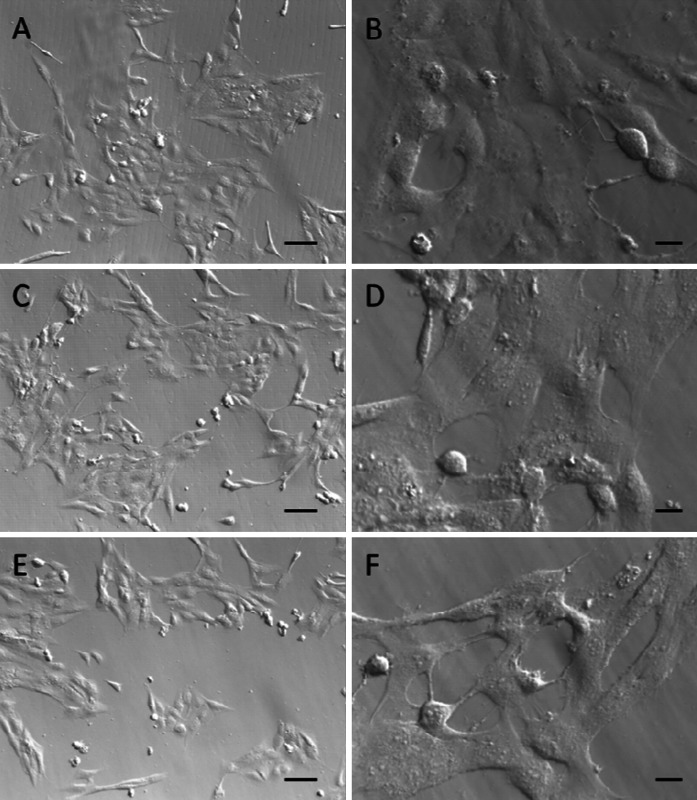
Microscopy images of HSkMEC cells treated with TbF_3_@CeF_3_ or TbF_3_@CeF_3_@SiO_2_ nanoparticles (*scale bars* at **a**, **c** and **e**: 40 μm, *scale bars* at **b**, **d** and **f**: 10 μm). **a** and **b**: control cells. **c** and **d**: cells treated with TbF_3_@CeF_3_ for 72 h, washed before imaging. **e** and **f**: cells treated with TbF_3_@CeF_3_@SiO_2_ for 72 h, washed before imaging

Potential effects of NCs on living cells were assessed in normal (HSkMEC) or cancer (B16F0) cell cultures in vitro. 72-h expositions of the cells to NCs had no effect on the amount of cells in comparison to the control, as revealed by SRB assays (Fig. 8). Our observation indicates that NCs have no toxic or inhibitory effects on the tested cells. Furthermore, cell morphology was also typical with extended protrusions, with no visible aberrations or defects, as shown by optical microscopy (Figs. 9, 10). Higher concentrations of NCs (0.5 mg/mL and higher) may cause cell damage and a reduced viability (data not shown). However, these concentrations are of low applicability due to lower stability of colloidal solutions.

## Conclusions

Luminescent nanoparticles based on TbF_3_@CeF_3_ can be synthesized by the co-precipitation method in Triton X-100 solution. During the nucleation and growth of nanocrystals, self-organization occurred resulting in the formation of the core/shell structure. These simultaneously formed core/shell nanocrystals were analysed by HR-TEM and EDX techniques what confirmed a complex character of the structures. The co-precipitation method allowed for obtaining small disks with an average diameter around 10 nm. The surface modification by SiO_2_ covering could be carried out by the described method. HR-TEM images showed that the precipitation process of discussed fluorides is complex and results. In the formation of NCs with a Tb^3+^-rich core and Ce^3+^-rich shell. The prepared fluoride NCs were used as the cores in the TbF_3_@CeF_3_@SiO_2_ structures. TEM images presented spherical shapes of NCs with a centrally placed fluoride core. Synthesized nanocrystals showed an intense green luminescence under UV irradiation what makes them good candidates as luminescent markers. Long luminescence lifetimes (2.52 and 3.53 ms) are other advantages of prepared NCs opening a possibility of time-resolved fluorescence lifetime imaging. 

TbF_3_@CeF_3_@SiO_2_ showed lower luminescence intensity than the cores due to the absorption of UV exciting radiation by the SiO_2_ shell and its quenching properties. The cell viability and proliferation test indicated that both types of the NCs were neutral for the investigated cells with no toxic or antiproliferative effects in the cell cultures, both for normal and for cancer cells. Furthermore, NCs had no visible effect on the cell morphology, as was shown by microscopy imaging. These results suggest safety and adequacy of NCs for future use in medical or biological applications. Covering NCs by SiO_2_ shell opens up new possibilities like modification of the surface of core/shell structures or chemical connection with important organic molecules and drugs.
